# Supplementary material for: The Enigmatic Role of TP53 in Germ Cell Tumours: Are We Missing Something?
Source: Int J Mol Sci. 2021 Jul 2;22(13):7160. doi: 10.3390/ijms22137160 (PMC8267694; doi:10.3390/ijms22137160)
Supplement: Supplementary file 1 [file ijms-22-07160-s001.zip › ijms-1237422-Supplementary.pdf]

Supplementary

Methods

Biopsy tissue samples of both TGCT and TC were collected. Hematoxylin-eosin slides were examined to ensure a content of neoplastic cells greater than 10%, the tumor areas were scraped. DNA extraction was performed with the GeneRead DNA FFPE Kit (Qiagen, Hilden, Germany) and a total of 10 ng of DNA was used for libraries' construction with the Ion AmpliSeq™Cancer Hotspot Panel v2, which included the most frequent mutations into the main 50 oncogenes and tumor suppressor genes involved in tumorigenesis. Emulsion PCR and chip loading were performed on the Ion Chef instrument and chip sequenced on the Ion Proton System. Sequencing data were processed with the Ion Torrent platform-specific pipeline software (Torrent Suite, V5.2.1). Moreover, Ion Reporter™ Software V5.2 and Integrative Genome Viewer software (http://www.broadinstitute.org/igv) were used for variant annotation and reads visualizations, respectively. The cut-off was set at 100× coverage, and a minimum mutant allele frequency of 5% was required to call non-synonymous variants in every sample.

Pyrosequencing was used for confirmation of the two pathogenic mutations detected in our cases; commercially available *KRAS* and *BRAF* Pyro Kits (QIAGEN) were used, which detected mutations in codons 12, 13, and 61 of the *KRAS* gene and in codons 600, 464, 469, of the *BRAF* gene. The Pyromark Q24 software was used for results analysis.
